# Opioids and constipation therapy in the last week of life: Their impact on patients, caregivers, and the location of death

**DOI:** 10.1097/MD.0000000000032718

**Published:** 2023-01-20

**Authors:** Hugo Ribeiro, Júlia Magalhães, Tatiana Cardoso, Isabel Chaves-Castro, Carla Lopes-Mota, Elisabete Costa, Patrícia Rocha, Luísa Lopes, Ângela Bouça, Cristina Pereira, José Paulo Andrade, Marília Dourado

**Affiliations:** a Community Support Team in Palliative Care – Group of Health Centers Gaia, Portugal; b Center for the Study and Development of Continuing and Palliative Care – Faculty of Medicine, University of Coimbra, Portugal; c Coimbra Institute for Clinical and Biomedical Research (ICBR) - Group of Environment Genetics and Oncobiology (CIMAGO), Faculty of Medicine of the University of Coimbra, Portugal; d Doctoral Program in Palliative Care at the Faculty of Medicine of the University of Porto, Portugal; e Department of Biomedicine – Unity of Anatomy, Faculty of Medicine of the University of Porto, Portugal; f Center for Health Technology and Services Research (CINTESIS), Porto, Portugal.

**Keywords:** caregivers, end of life care, palliative care, opioid, pain management, constipation

## Abstract

The use of opioids to control pain at the end of life may cause constipation, a symptom that can negatively influence the well-being of patients and caregivers. The main aim of this study was to evaluate the impact of constipation on symptomatic control and patients’ overall quality of life at this stage. A particular focus was placed on opioids. We also intended to investigate whether constipation and caregiver fatigue is related to the place of death (hospital vs home). The approach of 121 patients followed in 2021 in their last week of life by a home team specialized in palliative care was analyzed in an observational, retrospective, non-interventional study. The patients were followed up for an average of 39.7 days. A total of 82.6% wished to die at home, which occurred in 74% of the cases. The constipation prevention protocol reduced constipation by 55.1%. It seems that morphine is more related with constipation and tapentadol seems to reduce constipation induced by opioids. Patients tended to die in hospitals when their caregivers were exhausted; however, it was not possible to determine a cutoff point using the Zarit scale, which was used to assess caregiver burden. Constipation in the last week of life does not seem to influence the well-being of patients or their caregivers significantly and the individualization of intensive treatment of constipation is needed. Different opioids have different probabilities of causing adverse effects such as constipation. Future special support mechanisms can be created and activated for the most tired caregivers to avoid exhaustion and promote death at home, if that is the patient’s will.

**Key points**

- Opioids are widely used in the last week of life to control symptoms, such as pain and dyspnea.
- Constipation is one of the most common side effects of opioids.
- Constipation is one of the most common symptoms in the last days of life.
- It is known that constipation is challenging to treat in patients needing specialized palliative care.
- Several studies have reported different outcomes regarding the probability of opioid-causing constipation.
- There have been no studies on the importance of treating constipation in the last week of life.
- We do not know the importance of palliative care home teams in the last week of life for patients and caregivers and their wishes.
- We did not have any information about caregivers’ exhaustion and the probability of dying at the hospitals.
- In this study, we determined the importance of treating constipation when patients have symptoms during the last days of life. It seems that it is not as important as we thought and has no influence on the quality of life of patients and caregivers.
- We identified some advantages of specialized palliative care at home.
- We could also identify opioids with a higher probability of causing constipation and opioids with a lower probability of causing that symptom.
- We could not identify a cutoff point using the Zarit scale, but patients died at the hospital and not at home (as they wanted to) when caregivers were exhausted (with Zarit scale points of >20).

## 1. Introduction and objectives

More than 40 million people will need specialized palliative care every year around the world.^[1]^ Pain is one of the most common symptoms in patients in need of palliative care^[2,3]^ and is most frequent at the end of life.^[3]^ The use of opioids to control pain in these situations is not only necessary but also ordinary. Opioid therapy has constipation as a side effect,^[4]^ which is one of the most common symptoms in patients with palliative needs, particularly at the end of life.^[3]^ Keeping patients at home, in comfort, and accompanied by their loved ones is the best option, but it is necessary to create and support home teams to attend to them in all suffering dimensions.

The existence of home palliative care teams requires trained caregivers.^[5,6]^ Therefore, mental and physical health of caregivers is one of the main concerns of palliative care teams.^[6,7]^

Since opioids are often used to treat pain and constipation inevitably appears as a side effect,^[4]^ the main aim of this study was to assess whether constipation and its management have a significant impact on the control of other symptoms and on the quality of life of patients and caregivers in the last week of life. We also intended to evaluate the leading causes of patient death in palliative care and their place of death with this follow-up, aiming to assess whether the latter contradicts the trend of death in hospitals.

As secondary objectives, we aimed to evaluate whether there are opioids with less potential for constipation while maintaining the same effectiveness in controlling pain, and whether the protocol used for prevention and treatment of constipation, following the recommendations in the literature,^[8]^ has been effective in the control of this symptom. Finally, it also intends to identify a cutoff point between caregiver burden and place of death.

## 2. Materials and methods

This study collected data from the last days of life of 121 patients who died in 2021, followed by a home palliative care team in northern Portugal. This was an observational, retrospective, non-interventional study, using medical and nursing records made in the last 8 days of life of patients followed by the same home palliative care team.

Data collection was carried out by recording in a protected Microsoft Excel sheet, without transferring the identity of the patients. Following the General Data Protection Regulation, the files were eliminated after the end of the study and publication of the results.

The following variables were included: age, sex, primary diagnosis, time elapsed from the start of follow-up by the team until death (in days), place of death (home, hospital, or other), constipation for >3 days in the last 8 days of life, type of constipation (mechanical or functional), opioid and dose used, and laxative use.

The recommended therapy to prevent constipation included the use of first-line osmotic laxatives such as macrogol and lactulose when patients had been prescribed opioids on a fixed regimen and/or had a history of constipation.^[4,8,9]^ If >3 days of constipation, the patient started a bowel-stimulating laxative (such as bisacodyl).^[8]^ After >4 days of constipation, they began Senna and/or contact laxatives, such as sodium lauryl sulfate or sodium docusate.^[8]^ If the situation did not improve, a manual assessment was performed on the sixth or seventh day, and as there was no possibility of obstruction (we didn´t have any patient with mechanical constipation), a cleansing enema was performed.^[4,8]^

Whenever possible, the Edmonton scale was used to assess symptoms,^[10]^ the palliative outcome scale (POS) to assess patients’ quality of life,^[11]^ and the palliative performance scale to measure functionality.^[12]^ Caregiver burden was assessed using the Zarit scale.^[13]^

Qualitative variables were presented as absolute and relative frequencies and quantitative variables with means and standard deviations. The significance level for rejecting the null hypothesis was set at (α) ≤.05. Fisher exact test, Mann–Whitney U test, logistic regression, and receiver operating characteristic curves were used. The normality of distribution was analyzed using the Shapiro–Wilk test and the homogeneity of variances using the Levene test. The qualitative variables were transformed into dummy variables. The Hosmer–Lemeshow goodness-of-fit test was used for the goodness of fit for the logistic regression.

Statistical analysis was performed using Statistical Package for the Social Sciences software (version 28.0 for Windows, IBM Corp., Armonk, NY).

## 3. Results

In 2021, 198 patients were monitored by the same home palliative care team, of which 121 died (61%). Patients who died had a mean age of 78.1 years, between a minimum of 34 years and a maximum of 103 years, and 69 males (67%). They were all bedridden and totally dependent on daily living activities, with functionality measured by palliative performance scale^[12]^ between 10% and 30%.

The home palliative care team followed patients for an average of 39.7 days, but cancer patients were followed up for a mean of 47 days (20.5% more follow-up time).

**Figure 1.**
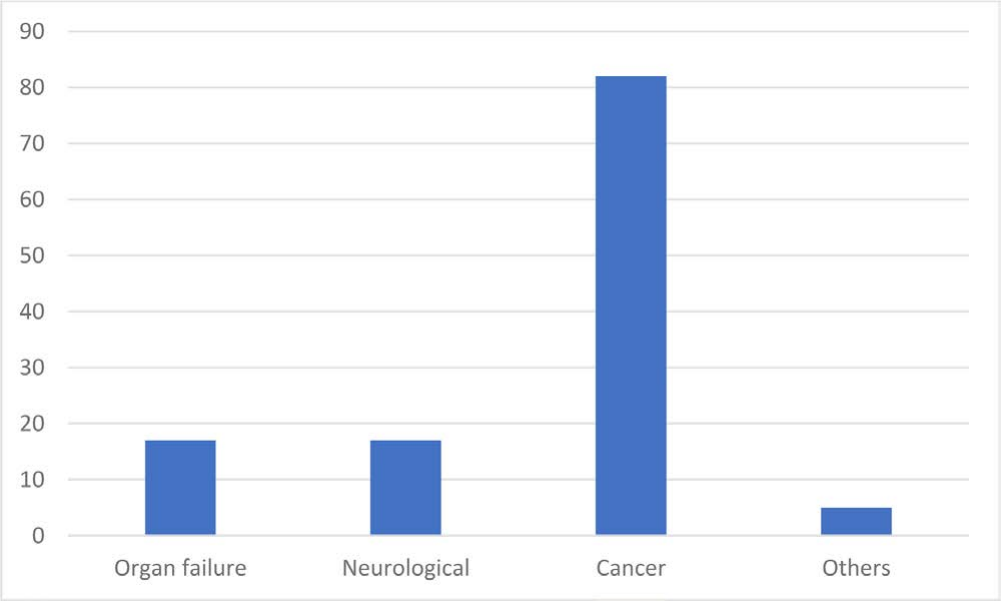
Causes of death (absolute numbers). Causes of death: cancer (n = 81), neurological diseases (n = 17), organ failure (n = 17), and others (n = 6).

As shown in Figure 1, cancer (66.9%, n = 81) was the most frequent cause of death in this period, followed by neurological diseases (14%), organ failure (14%), and other causes (4.2%), which included autoimmune diseases, chronic pain syndromes, and frailty syndrome in the elderly.

**Figure 2.**
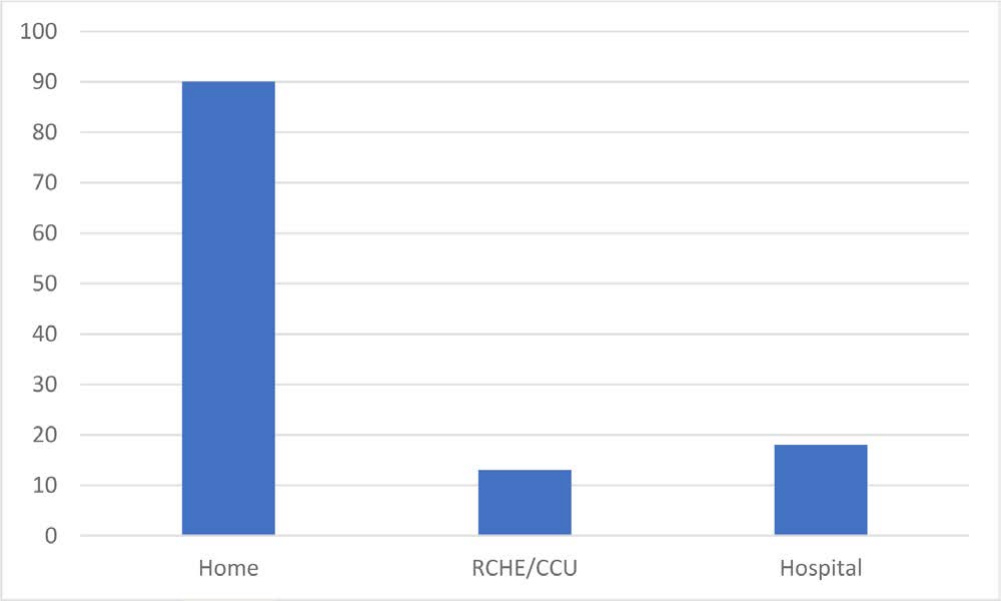
Place of death (absolute number). Place of death: home (n = 90), residential care homes for the elderly/continuing care units (ERPI/UCC [n = 13], and hospital [n = 18]).

Of the 121 patients, 100 registered their domicile as the intended place of death (82.6%). As observed in Figure 2, approximately 74% of the patients died at home (n = 90), 15% in the hospital (n = 18), and 11% in institutions (residential care homes for the elderly and continuing care unit).

Of the 121 patients who died during the study period, no patient had mechanical constipation, and 95.9% (n = 116) had opioids prescribed in the last week of life; of these, 53.7% (n = 65) did not have constipation during this week.

On average, patients had a score of 28.9 in the last week of life on the Edmonton scale^[10]^ and a POS score^[11]^ of 17.5.

Regarding the degree of caregiver burden, the mean score obtained using the Zarit scale was 40, and the main caregivers thus presented a moderate to severe burden on average. Regarding the impact of constipation in the last week of life on caregivers, it was observed that they were less tired (average Zarit score of 38).

However, no significant relationships were found between place of death, caregiver burden, or Edmonton scale score and constipation (*P* > .05), as shown in Table 1.

**Table 1:** Bivariate analysis.

	Home	Hospital	Sig.
Obstipation			.531
No	51 (77.3%)	15 (22.7%)	
Yes	39 (70.9%)	16 (29.1%)	
Zarit^[14]^ (M; SD)	20.72 (7,49)	17.64 (14,53)	.713
Edmonton^[12]^ (M; SD)	30.29 (14.03)	24.93 (10.85)	.140

M = mean, SD = standard deviation, Sig. = significance.

Then, a logistic regression was performed with the variables Edmonton, Zarit and constipation as independent or predictive variables and the local variable of death as the explained or dependent variable. The difference between the model plus the independent variables and the model with the constant alone was not statistically significant (*χ*^2^ (3) = 5.809, *P* = 121). The model explains 7.8% of the decrease in uncertainty in identifying the place of death. The Hosmer–Lemeshow goodness-of-fit test was *χ*^2^ (8) = 21.174, *P* = .007, indicating a poor fit to the data. The independent variables were not significant predictors of place of death (*P* > .05), as shown in Table 2.

**Table 2:** Logistic regression (place of death).

		B	SE	Wald	Sig.	Exp (B)	95% CI for Exp (B)	
							Lower	Upper
Step	Edmonton	−.031	.020	2.369	.124	.970	.933	1.008
	Zarit	−.019	.024	.645	.422	.981	.935	1.028
	Obstipation	.628	.479	1.718	.190	1.874	.733	4.792
	Constant	−.335	.683	.241	.624	.715		

B = coefficient for the constant in the null model, CI = confidence interval, Exp (B) = exponentiation of the B coefficient, SE = standard error, Sig. = significance. Wald = Wald test.

Regarding the use of opioids, 95 patients were prescribed morphine, of which 42 were also on another major opioid, 25 were on fentanyl, 20 were on tapentadol, 13 were on buprenorphine, and 5 were on tapentadol and buprenorphine.

The occurrence of constipation in relation to the use of different opioids varied, as shown in Table 3.

**Table 3:** Frequency of constipation in the last week of life with the different opioids.

Opioid	Patients with constipation (%)
Morphine	46 (48.4%)
Morphine + another opioid	17 (40.5%)
Fentanyl	11 (44%)
Buprenorphine	6 (46.2%)
Tapentadol	6 (30%)
Tapentadol + buprenorphine	2 (40%)

On average, 41.5% of the patients who had constipation in the last week of life were taking opioids.

The significant relationships between the variables under study and constipation are listed in Table 4.

**Table 4:** Significant relationship between constipation and the other variables under study.

	No		Yes		
	N	%	N	%	Sig.
Morphine	23	51.1%	22	48.9%	.577
Tapentadol_50_100	3	42.9%	4	57.1%	.701
Tapentadol100_200	0	0.0%	1	100.0%	.455
Tapentadol200_400	0	0.0%	0	0.0%	–
Buprenorphine17.5_35	3	60.0%	2	40.0%	1.000
Buprenorphine52.5	0	0.0%	1	100.0%	.455
Buprenorphine70	1	25.0%	3	75.0%	.329
Fentanyl12.5	5	35.7%	9	64.3%	.160
Fentanyl25	8	47.1%	9	52.9%	.602
Fentanyl50	2	22.2%	7	77.8%	.077
Fentanyl75	2	40.0%	3	60.0%	.658
Fentanyl100	0	0.0%	1	100.0%	.455
Lactulose	1	7.7%	12	92.3%	.001***
Macrogol	3	75.0%	1	25.0%	.625
Macrogol + Sodium bicarbonate	13	34.2%	25	65.8%	.003**
Sodium picosulfate	2	10.5%	17	89.5%	.001***
Isacodyl	6	24.0%	19	76.0%	.001***
Microlax	1	5.0%	19	95.0%	.001***
Senna	2	18.2%	9	81.8%	.022*
Cleansing enema	0	0.0%	3	100.0%	.091
Manual	0	0.0%	4	100.0%	.040*

N = absolute number of patients, Sig. = significance.

* P ≤ .05.

** P ≤ .01.

*** P ≤ .001.

Patients treated with morphine seem to have more constipation and patients treated with tapentadol seem to have less constipation, according with other studies^[14–17]^ that had less patients.^[16,17]^ In patients treated with the remaining opioids, there were no significant differences in developing constipation.

Regarding caregiver burden, it was not possible to define a cutoff point for the place of death. The area under the receiver operating characteristic curve of sensitivity and specificity between the place of death and caregiver burden was not statistically significant (*P* = .713), there was no reason to find a cutoff point. However, all patients who died in the hospital during the study period had caregivers with a Zarit score^[13]^ ≥20.

## 4. Discussion and conclusions

Data related to the primary diagnosis and cause of death showed that most patients had cancer (67.8%). Despite this, non-cancer patients represent the majority (approximately 70%) of patients in need of palliative care, worldwide.^[1]^ The present results suggest that patients with non-cancer diseases have increased difficulty in accessing palliative care, which is reinforced by the fact that the follow-up time of patients with cancer is 20.5% longer than the mean follow-up time of all patients. Overall, these facts suggest that cancer patients are referred earlier to palliative care and with that, the opportunity for greater satisfaction with the response to their needs and the care provided.

The fact that a significant number of patients expressed a wish to die at home (100 out of 121), which happened to the majority in this study, reinforces the benefits of palliative care at home since, in Portugal, only 29.6% of patients die at home.^[18]^ However, only 8.9% referred to the hospital as their preferred place of death in a country where 61.7% of people die in these institutions.^[18]^

There are published papers of varying quality, as reviewed by Hofmeister and colleagues, assessing different components of palliative care in the home interventions and measuring different outcomes.^[19]^ To be meaningful to patients, these interventions need to be consistently evaluated with outcomes that matter to the patients and caregivers,^[19]^ as we presented in this work.

Morphine appeared to be the opioid that is more related with constipation. In contrast, tapentadol appears to be less associated with constipation.

Constipation occurs in approximately 97% of patients on chronic opioid therapy.^[9]^ The protocol followed by the home palliative care team significantly reduced constipation by 55.1% in the studied patients.^[4,8,9]^

Constipation in the last week of life does not seem to influence the well-being of patients, which is noticeable by the lack of statistical significance between Edmonton and POS of constipated and non-constipated patients. Constipation also had no statistically significant influence on caregiver burden. Thus, intensive therapies to control this symptom in the last week of life should be considered only in specific cases in which the general condition worsens.

Although we cannot present a significant cutoff point between the caregiver burden measured by the Zarit scale and the place of death with high sensitivity and specificity, it is evident that patients tend to die in the hospital when the caregiver is most exhausted.

In this sense, it is important to develop special support mechanisms for caregivers who present with symptoms and signs of tiredness to fulfill the wishes of both (patients and caregivers) to enable their end of life at home.

This work has limitations, not only because it is a unicentric study, but also because of the small sample size. However, to our knowledge, this is the first study to interpret the relationship between the use of opioids, laxatives, constipation, caregiver burden, and the location of death. We can now understand that some symptoms, such as constipation, may have a more conservative approach at the end of life, ensuring the well-being of both patients and caregivers. More similar studies involving larger numbers of patients are needed to obtain additional data and solid conclusions.

## Author contributions

**Conceptualization:** Hugo Ribeiro, Marília Dourado.

**Data curation:** Hugo Ribeiro, Tatiana Cardoso, Isabel Chaves-Castro, Elisabete Costa, Patrícia Rocha.

**Formal analysis:** Hugo Ribeiro, Elisabete Costa, Patrícia Rocha, Cristina Pereira.

**Funding acquisition:** Hugo Ribeiro.

**Investigation:** Hugo Ribeiro, Tatiana Cardoso, Isabel Chaves-Castro, Ângela Bouça, Cristina Pereira.

**Methodology:** Hugo Ribeiro, Elisabete Costa, Cristina Pereira.

**Project administration:** Hugo Ribeiro, Elisabete Costa.

**Resources:** Hugo Ribeiro.

**Software:** Hugo Ribeiro, Patrícia Rocha, Ângela Bouça.

**Supervision:** Hugo Ribeiro, Carla Lopes-Mota, José Paulo Andrade, Marília Dourado.

**Validation:** Hugo Ribeiro, Júlia Magalhães, Carla Lopes-Mota, Luísa Lopes, Ângela Bouça, José Paulo Andrade, Marília Dourado.

**Visualization:** Hugo Ribeiro, Júlia Magalhães, Carla Lopes-Mota, Luísa Lopes, José Paulo Andrade, Marília Dourado.

**Writing – original draft:** Hugo Ribeiro, Júlia Magalhães, Tatiana Cardoso, José Paulo Andrade, Marília Dourado.

**Writing – review & editing:** Hugo Ribeiro, Júlia Magalhães, Tatiana Cardoso, Isabel Chaves-Castro, Carla Lopes-Mota, Luísa Lopes, José Paulo Andrade, Marília Dourado.

## References

[1] Assessing national capacity for the prevention and control of noncommunicable diseases: report of the 2019 global survey. Geneva: World Health Organization. 2020.

[2] Kreher M. Symptom control at the end of life. Med Clin N Am. 2016;100:1111–22. 27542430 10.1016/j.mcna.2016.04.020

[3] Gill TM Gahbauer EA Han L. Trajectories of disability in the last year of life. N Engl J Med. 2010;362:1173–80. 20357280 10.1056/NEJMoa0909087 PMC2877372

[4] Neefjes ECW van der Wijngaart H van der Vorst MJDL. Optimal treatment of opioid induced constipation in daily clinical practice – an observational study. BMC Palliat Care. 2019;18:31. 30922276 10.1186/s12904-019-0416-7 PMC6439982

[5] Ahlner-Elmqvist M Jordhøy MS Bjordal K. Characteristics and quality of life of patients who choose home care at the end of life. J Pain Symptom Manage. 2008;36:217–27. 18400462 10.1016/j.jpainsymman.2007.10.010

[6] Alonso-Babarro A Bruera E Varela-Cerdeira M. Can this patient be discharged home? factors associated with at-home death among patients with cancer. J Clin Oncol. 2011;29:1159–67. 21343566 10.1200/JCO.2010.31.6752 PMC3083871

[7] Alonso-Babarro A Astray-Mochales J Domínguez-Berjón F. The association between in-patient death, utilization of hospital resources and availability of palliative home care for cancer patients. Palliat Med. 2013;27:CD003448. 10.1177/0269216312442973 22492481

[8] Candy B Jones L Larkin PJ. Laxatives for the management of constipation in people receiving palliative care. Cochrane Database Syst Rev. 2015;2015:68–75. 10.1002/14651858.CD003448.pub4 PMC6956627 25967924

[9] LoCasale RJ Datto C Wilson H. The burden of opioid-induced constipation: discordance between patient and health care provider reports. J Manag Care Spec Pharm. 2016;22:236–45. 27003553 10.18553/jmcp.2016.22.3.236 PMC10397844

[10] Chang VT Hwang SS Feuerman M. Validation of the edmonton symptom assessment scale. Cancer. 2000;88:2164–71. 10813730 10.1002/(sici)1097-0142(20000501)88:9<2164::aid-cncr24>3.0.co;2-5

[11] Antunes B Ferreira PL. Validation and cultural adaptation of the Integrated Palliative care Outcome Scale (IPOS) for the Portuguese population. BMC Palliat Care. 2020;19:178. 33234116 10.1186/s12904-020-00685-z PMC7687758

[12] Olajide O Hanson L Usher BM. Validation of the palliative performance scale in the acute tertiary care hospital setting. J Palliat Med. 2007;10:111–7. 17298259 10.1089/jpm.2006.0125

[13] Seng BK Luo N Ng WY. Validity and reliability of the zarit burden interview in assessing caregiving burden. Ann Acad Med Singap. 2010;39:758–63. 21063635

[14] Kon R Ikarashi N Hayakawa A. Morphine-induced constipation develops with increased Aquaporin-3 expression in the colon via increased serotonin secretion. Toxicol Sci. 2015;145:337–47. 25766885 10.1093/toxsci/kfv055

[15] Nelson AD Camilleri M. Opioid-induced constipation: advances and clinical guidance. Ther Adv Chronic Dis. 2016;7:121–34. 26977281 10.1177/2040622315627801 PMC4772344

[16] Kwong WJ Hammond G Upmalis D. Bowel function after tapentadol and oxycodone immediate release (IR) treatment in patients with low back or osteoarthritis pain. Clin J Pain. 2013;29:664–72. 23835764 10.1097/AJP.0b013e318274b695

[17] Mark EB Frøkjær JB Hansen TM. Although tapentadol and oxycodone both increase colonic volume, tapentadol treatment resulted in softer stools and less constipation: a mechanistic study in healthy volunteers. Scand J Pain. 2021;21:406–14. 33606931 10.1515/sjpain-2020-0151

[18] Gomes B Sarmento VP Ferreira PL. [Epidemiological study of place of death in Portugal in 2010 and comparison with the preferences of the Portuguese population]. Acta Med Port. 2013;26:327–34. 24016640

[19] Hofmeister M Memedovich A Dowsett LE. Palliative care in the home: a scoping review of study quality, primary outcomes, and thematic component analysis. BMC Palliat Care. 2018;17:41. 29514620 10.1186/s12904-018-0299-z PMC5842572

